# WT1 and Cyclin D1 Immunohistochemistry: A Useful Adjunct for Diagnosis of Pediatric Small Round Blue Cell Tumors on Small Biopsies

**DOI:** 10.3390/diagnostics11122254

**Published:** 2021-12-02

**Authors:** Lucia Salvatorelli, Rosalba Parenti, Giuseppe Broggi, Giada Maria Vecchio, Giuseppe Angelico, Lidia Puzzo, Andrea Di Cataldo, Vincenzo Di Benedetto, Rita Alaggio, Gaetano Magro

**Affiliations:** 1 Department of Medical and Surgical Sciences and Advanced Technologies, “G. F. Ingrassia”, Anatomic Pathology, University of Catania, 95123 Catania, Italy; giuseppe.broggi@gmail.com (G.B.); giadamariavecchio@gmail.com (G.M.V.); giuangel86@hotmail.it (G.A.); lipuzzo@unict.it (L.P.); g.magro@unict.it (G.M.); 2 Department of Biomedical and Biotechnological Sciences, Physiology Section, University of Catania, 95123 Catania, Italy; parenti@unict.it; 3 Pediatric Hematology and Oncology Unit, Department of Pediatrics, University of Catania, 95123 Catania, Italy; adicata@unict.it; 4 Pediatric Surgery Unit, Department of Medical and Surgical Sciences and Advanced Technologies, “G. F. Ingrassia”, University of Catania, 95123 Catania, Italy; vdb@chirpedunict.it; 5 Pathology Unit, Bambino Gesù Children’s Hospital, IRCCS, 00165 Rome, Italy; rita.alaggio@opbg.net

**Keywords:** immunohistochemistry, WT1, cyclin D1, small round blue cell tumors, small biopsy

## Abstract

Pediatric small round blue cell tumors (SRBCTs) are a heterogeneous group of neoplasms with overlapping morphological appearance. Accordingly, their diagnosis is one of the most difficult in the field of surgical pathology. The most common tumors include rhabdomyosarcoma, Ewing’s sarcoma, neuroblastoma, lymphoblastic lymphoma and Wilms’ tumor (the blastemal component). Over time their diagnosis has become more difficult due to the increasing use of small biopsies. However, the advent of immunohistochemistry has improved the quality of diagnosis in most cases by the application of an adequate panel of immunomarkers. Recently, WT1 and Cyclin D1 have been shown to be useful in the differential diagnosis of SRBCTs on surgically-resected specimens, showing a diffuse cytoplasmic positivity of the former in all RMSs and a diffuse nuclear staining of the latter in both EWS and NB. The aim of the present study was to investigate the expression of WT1 and Cyclin D1 on small biopsies from a series of 105 pediatric SRBCTs to evaluate their diagnostic utility. Both immunomarkers were differentially expressed, with a diffuse and strong cytoplasmic staining for WT1 limited to all cases of RMS, and a diffuse nuclear staining for cyclin D1 restricted to all cases of EWS and NB. Notably, the expression of WT1 and cyclin D1 was also retained in those cases in which the conventional tumor markers (myogenin, desmin and MyoD1 for RMS; CD99 for EWS; NB84 for NB) were focally expressed or more rarely absent. The present study shows that WT1 and Cyclin D1 are helpful immunomarkers exploitable in the differential diagnosis of pediatric SRBCTs on small biopsies, suggesting their applicability in routine practice.

## 1. Introduction

Pediatric small round blue cell tumors (SRBCTs) are a heterogeneous group of neoplasms which include rhabdomyosarcoma (RMS), Ewing’s sarcoma (EWS), neuroblastoma (NB), lymphoblastic lymphoma (LL) and Wilms’ tumor. These neoplasms may show overlapping morphological and sometimes immunohistochemical features [1,2,3,4,5,6]. In daily practice, these tumors are often diagnosed on small biopsies, making diagnosis more difficult, especially if the pathologist is not familiar with these lesions or if tumors show unusual morphology and/or immunohistochemical profile and/or clinical presentation [2,4,7,8,9]. Currently, the diagnosis of SRBCTs is based on the combined evaluation of the morphological, immunohistochemical and cytogenetic/molecular features [1,2,3,4,5,6]. As an appropriate diagnostic approach is mandatory to assure a correct prognostic information and proper treatment, the identification of novel sensitive and specific immunomarkers, easily available and applicable also in developing countries, seems to be crucial.

The WT1 gene is a transcription factor essential for normal development of the urogenital system and encodes for Wilms tumor protein located on chromosome 11p13. It has different functions, including developmental, tumor suppressor and oncogenic properties [10,11,12,13,14], due to the production of various isoforms of WT1 resulting from an alternative splicing [15,16,17,18]. The WT1 protein can be variably demonstrated by means of immunohistochemistry in fetal, adult and neoplastic tissues, i.e., exclusively nuclear, cytoplasmic staining or both, according to the antibodies used (anti-C or N-terminus WT1 protein) [19,20,21,22,23,24,25,26]. Cyclin D1, also known as BCL1 and encoded by BCL1/PRAD1 gene, is responsible for transition to S phase by phosphorylating the retinoblastoma gene product, which releases transcription factors to initiate DNA replication. Its overexpression promotes transformation into a malignant phenotype, including carcinomas, sarcomas and lymphomas [1,27]. Several genomic alterations such as chromosomal amplification or translocation, post-transcriptional regulation or post-translational protein stabilization have been observed [28,29,30]. Recently, our research group has demonstrated the utility of both WT1 and cyclin D1 in the differential diagnosis of SRBCTs on surgically-resected specimens, the former being diffusely expressed in all subtypes of RMS and the latter in EWS and NB [20,31,32,33,34,35]. Based on these results, we aimed to asses if both WT1 and Cyclin D1 can be exploitable as a useful adjunct in the diagnosis of SRBCTs on small biopsies. Accordingly, a large series of 105 small biopsies from pediatric SRBCTs, including RMS, EWS, NB, Wilms’ tumor and LL, were immunohistochemically investigated. 

## 2. Materials and Methods

The cases were retrospectively retrieved from the surgical pathology archives of the Anatomic Pathology, Department of Medical, Surgical, and Advanced Technologies (G.F. Ingrassia) at the University of Catania. Clinical data were obtained from the original pathology reports. Hematoxylin and eosin (H&E)–stained slides and a variable number of slides stained with several antibodies were available for each case. In addition, at least 1 representative formalin-fixed, paraffin-embedded block was available for each case. All the H&E slides were reviewed by one expert surgical pathologist (R.A. and G.M.) of the Italian Group of Oncologic Pediatric Pathology (GIPPI), and the diagnoses were histologically and immunohistochemically confirmed using the current well established criteria (Table 1) [36,37,38,39,40]. The diagnoses rendered on small biopsies were confirmed in some resected tumors after chemotherapy (3 cases of EWS; 12 cases of NB; 2 cases of alveolar RMS; 1 case of embryonal RM; 1 case of spindle cell/sclerosing RM) by means of histology and immunohistochemistry. Molecular data (fusion gene products) by reverse-transcription polymerase chain reaction (RT-PCR) were also available for the following tumors diagnosed on small biopsies (Table 1): (i) all cases of EWS; 13 out of 14 cases showed *EWS/FL*I1 fusion from t(11;22) (q24; q12), with the remaining case harboring *EWS/ERG* from t(21;22;) (q24; q12); (ii) 6 out 14 cases of alveolar RMS; all six cases showed (*PAX3/FOX01* fusion from t(2:13) (q35; q14); (iii) 10 cases of embryonal RMS; all tumors did not harbor the *PAX3/FOX01* or *PAX7/FOX01* fusions; (iv) one out of three cases of spindle cell/sclerosing RMS harbored *MYOD1 L122R* mutation. 

The following tumors were selected: (i) 14 cases of extra-skeletal EWS (two cases in the abdomen, four cases in the pelvis, two cases in the skull, two cases in the mediastinum, two cases in the lower limbs, one case of bone metastasis and one case at para-spinal level); the patients were predominantly males (eight out of 14 cases) and 11 out of 14 cases were children aged 10–21 years; (ii) 33 cases of RMS, including 16 cases of embryonal RMS (three cases in the abdominal site, four cases in the pelvic site, three cases in the hard palate, one case in bone marrow, one case in the oro-pharynx, one case in the orbit and one case in the testis), 14 cases of alveolar RMS (three cases in abdominal site, two cases in the upper limbs, one case in the lower limbs, one case in the palate, three cases of bone metastases, three cases in the pelvic region, one case in the testis, one case in the orbit and one case in the breast), three cases of spindle cell/sclerosing RMS (two cases in the oral cavity; one case in the paratesticular region); the incidence of RMS was higher in males (22 out of 33 cases) than in females (11 out of 33 cases), of which 18 cases were between 0–5 years, seven between 5–10 years, six between 10–15 years and two between 15–21 years; (iii) 44 cases of NB (20 cases in abdominal site, seven cases of bone marrow metastases, three cases of lymph node metastases, five cases in the adrenal gland, six cases of bone metastases, one case in mediastinal site, 2twocases in subcutaneous tissue); the incidence was higher in males (28 out of 44 cases); most tumors (31 out of 44) occurred in children aged 0–5 years, 10 cases between 6–10 years and three cases between 10–15 years; (iv) 11 cases of LL, including 7 cases of T-cell precursor (six cases in the mediastinum and one case in the palate) and four cases of the B-cell precursor variant (two cases in the testis, one case in the mediastinum and one case in the abdominal region); most tumors occurred in males (seven out of 11 cases) with an age ranging from 10–21 years (seven out of 11 cases); (v) three cases of renal Wilms’ tumors affected females with an age of one, five and seven years, respectively.

Immunohistochemical analyses were performed as previously reported in detail [20,32]. Briefly, after appropriate deparaffinization and pretreatments, sections were incubated with anti–Cyclin D1 (SP4; NeoMarkers, Fremont, CA, USA; prediluted antibody) and WT1 (clone WT 6F-H2) (Dako, Glostrup, Denmark) at pH 6.0 for 60 min at room temperature. Microwave pretreatment was crucial to enhance the staining in all examined samples. Accordingly, all sections were pretreated with citrate buffer (pH 6.0) and exposed to radiation in a microwave oven. To reduce the commonly seen nonspecific immunoreactivity due to endogenous biotin, sections were pretreated with 10 mg/mL of ovalbumin in phosphate-buffered saline followed by 0.2% biotin in phosphate-buffered saline, each for 15 min at room temperature. The bound antibody was revealed by incubation with 3,3′-diaminobenzidine (Sigma-Aldrich, St. Louis, MO, USA) in 0.01% H_2_O_2_ for 5 min at room temperature. Sections were then counterstained with hematoxylin, dehydrated, and mounted. Negative controls, involving the omission of the primary antibody, were included. The percentage of positively stained cells was assessed by semiquantitative optical analysis according to a 4-tiered system (<1% positive cells = negative staining; 1–10% positive cells = focal staining; 11–50% positive cells = heterogeneous staining; 50% positive cells = diffuse staining). Staining intensity was graded as weak, moderate or strong intensity.

## 3. Results

Immunohistochemical results are summarized in the Table 2. 

**Figure 1:**
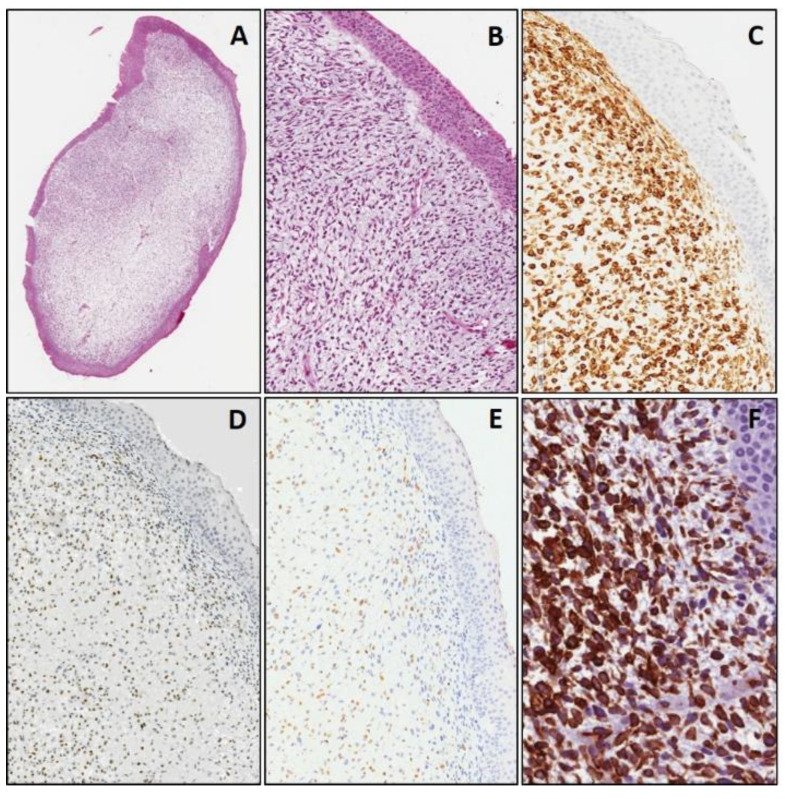
Embryonal rhabdomyosarcoma, botryoid variant. Neoplastic cells beneath epithelial layer, at low (**A**) and higher (**B**) magnification, showing diffuse positivity for myogenic markers: desmin (**C**), myogenin (**D**) and MyoD1 (**E**). Diffuse and strong cytoplasmic staining of neoplastic cells for WT1 (**F**). ((**A**): original magnification 40×; (**B**–**E**): original magnification 100×; (**F**): original magnification 200×).

Among SRBCTs, all cases (33/33) of RMS, regardless of the histologic subtypes (embryonal, alveolar or spindle cell/sclerosing), showed at least two of the three myogenic markers (desmin, myogenin and MyoD1) variably expressed in terms of extension (Figure 1), along with no staining for CD99, NB84 and TdT. Three cases of alveolar RMS also showed diffuse CD56 immunostaining. 

**Figure 2:**
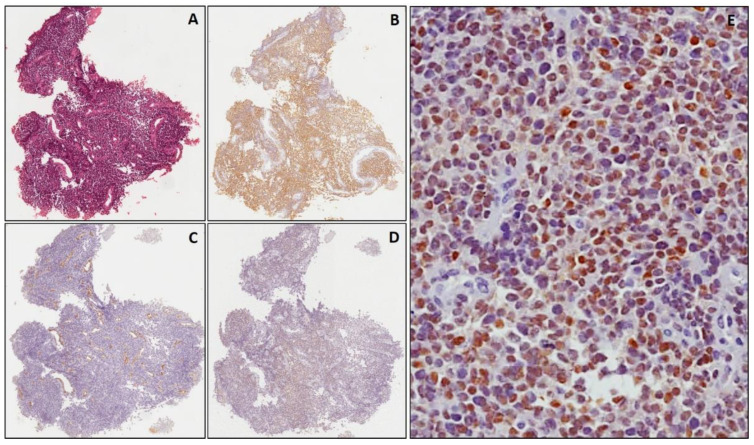
Ewing’s sarcoma, classic type. Serial sections from a small biopsy stained with hematoxylin & eosin (**A**). Neoplastic cells are diffusely positive for CD99 (**B**) and negative for WT1 (**C**); endothelial cells of intratumoral blood vessels are stained (internal control staining). In (**D**,**E**) diffuse nuclear staining for cyclin D1, at low and higher magnification, respectively. ((**A**–**D**): original magnification 40×; (**E**): original magnification 200×).

**Figure 3:**
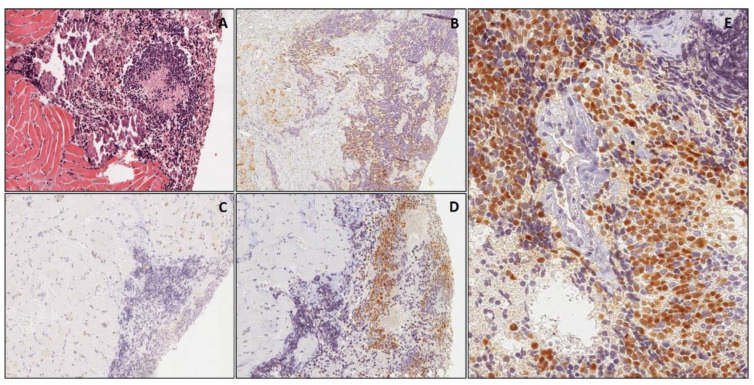
Poorly differentiated neuroblastoma A small biopsy from neuroblastoma infiltrating skeletal muscle, stained with hematoxylin & eosin (**A**). Neoplastic cells are diffusely positive for NB84 (**B**) and negative for WT1 (**C**). In (**D**,**E**), diffuse nuclear staining for cyclin D1, at low and higher magnification, respectively. ((**A**–**D**): original magnification 80×; (**E**): original magnification 200×).

All cases of EWS exhibited CD99 expression (Figure 2), with the exception of 2 cases in which a heterogeneous immunostaining as well as a non-specific focal staining of tumor stroma was observed. None of the cases was positive for myogenic markers, NB84, CD56 and TdT. All cases but one poorly differentiated NB exhibited immunostaining for NB84, which was diffuse in all but two cases in which only a focal immunoreactivity was obtained (Figure 3). In addition, CD56 was also co-expressed in all cases. No staining was observed for the myogenic markers, CD99 and TdT. All cases of LLs (both precursor T- and B- cells) were diffusely positive for TdT and other specific lineage markers, including T-cell markers (CD3, CD2, CD5, CD7) and B-cell markers (PAX 5, CD79a and CD19). All the T-cell precursor cell LLs were also CD99-positive. No staining was detected for the myogenic markers, NB84 and CD56. All cases of Wilms’ tumor showed heterogeneous to diffuse cytoplasm/nuclear expression of WT1 in both the epithelial and blastemal component; a diffuse immunostaining for CD56 was also documented in the blastemal component.

**Figure 4:**
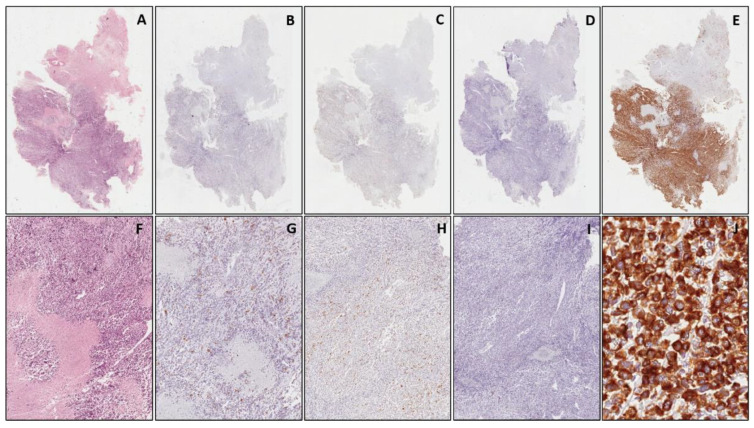
Embryonal rhabdomyosarcoma. Serial sections from a small biopsy, stained with hematoxylin & eosin at low (**A**) and higher (**F**) magnification. Neoplastic cells show focal expression of desmin ((**B**,**G**), at low and higher magnification, respectively) and myogenin ((**C**,**H**) at low and higher magnification, respectively) while no staining for MyoD1 ((**D**,**I**), at low and higher magnification, respectively). In (**E**) (low magnification) and (**J**) (higher magnification), diffuse and strong cytoplasmic expression of WT1. ((**A**–**E**): original magnification 40×; (**F**–**I**): original magnification 100×; (**J**): original magnification 200×).

As far as WT1 immunohistochemical expression is concerned, a diffuse and strong cytoplasmic staining (>90% positive cells) was detected in all cases of RMS, regardless of the histologic subtypes (Figure 1). Notably, the WT1-positive cases also included two cases of alveolar and three cases of embryonal RMS which showed focal expression of desmin and myogenin along with no staining for MyoD1 (Figure 4). The diagnosis of the two above mentioned cases of alveolar RMS was confirmed by means of molecular biology (*PAX3/FOX01* fusion). The remaining other SRBCTs, including EWS, NB and LL, were negative for WT1. As expected, none of the tumors tested showed nuclear immunostaining for WT1, except for Wilms’ tumors (three out of three cases) in both epithelial and blastemal component, in addition to a cytoplasmic immunostaining. In all cases of SRBCTs WT1 variably stained the cytoplasm of endothelial cells of intra-tumoral blood vessels and served as internal control. 

**Figure 5:**
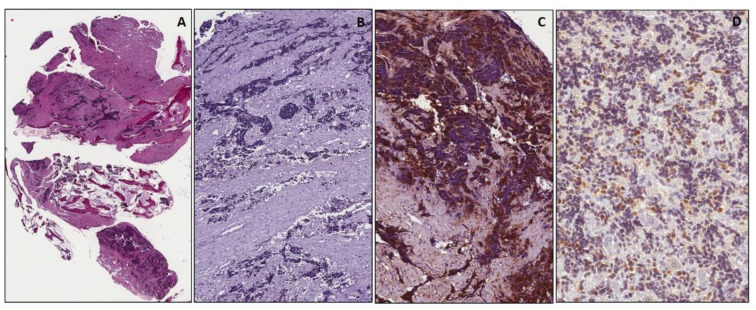
Ewing’s sarcoma confirmed by molecular analysis for *EWSR1-FLI1* fusion. Small biopsy stained with hematoxylin & eosin (**A**); the diagnosis was confirmed by molecular analysis (*EWSR1-FLI1* fusion). In (**B**,**C**), two different areas of the same tumor showing, respectively, no immunostaining and positivity for CD99 not only restricted to neoplastic cells but also focally extending into tumor stroma (non-specific immunostaining due to sampling artefacts). Neoplastic cells showing diffuse nuclear staining for cyclin D1 (**D**). ((**A**) original magnification 40×; (**B**,**C**): original magnification 100×; (**D**): original magnification 200×).

**Figure 6:**
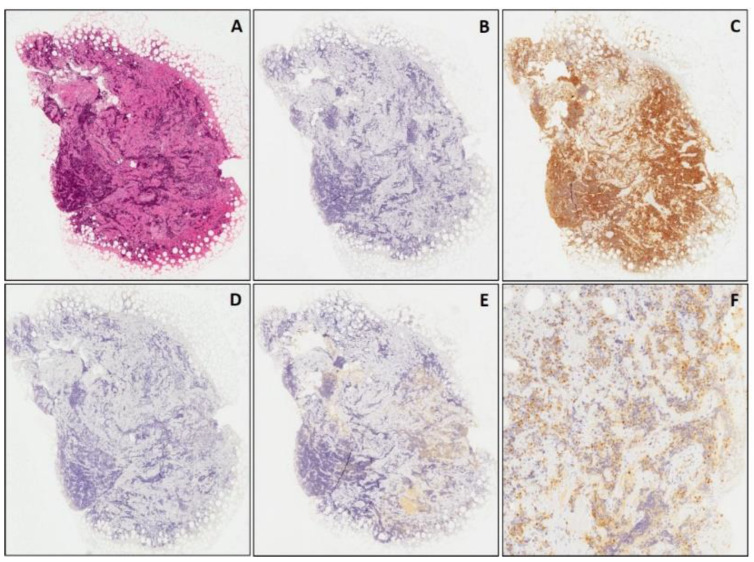
Subcutaneous nodule in a patient with a previous diagnosis of neuroblastoma. (**A**) Low magnification of a subcutaneous nodule of NB: section stained with hematoxylin & eosin. Neoplastic cells were negative for NB84 (**B**) and WT1 (**D**); diffuse staining was obtained with CD56 (**C**) and cyclin D1, at low (**E**) and higher (**F**) magnification. ((**A**–**E**): original magnification 40×; (**F**): original magnification 100×).

As far as cyclin D1 is concerned, all cases of EWS (14/14) displayed a strong and diffuse nuclear expression (>50% positive cells) (Figure 2), including two cases in which the immunostaining for CD99 was not only heterogeneous but also focally extended into tumor stroma (non-specific immunostaining) due to sampling artefacts (Figure 5). The diagnosis of these two cases was confirmed by molecular evidence of *EWS/FLI1* fusion. Similarly, all cases (44 out of 44) of poorly differentiatedNB showed a diffuse (>70% positive cells) nuclear expression of cyclin D1 (Figure 3), including three cases in which NB84 was lacking (one metastatic subcutaneous case with a previous diagnosis of poorly differentiated NB) (Figure 6) or only focally expressed (two cases histologically and immunohistochemically confirmed in the surgically-resected specimen after chemotherapy). No immunoreactivity for cyclin D1 was observed in the other SRBCTs, including RMS (regardless of subtype), LL and Wilms’ tumor. In all cases of SRBCTs, cyclin D1 variably stained the nuclei of endothelial cells of intra-tumoral blood vessels and served as internal control.

## 4. Discussion

Differential diagnosis of pediatric SRBCTs is challenging due to the overlapping morphological and immunohistochemical features. In addition, their clinical presentation can be equivocal as radiological imaging is not specific and it is not always possible to distinguish the exact tumor site of origin (difficulties in establishing the origin from the kidney, adrenal gland or soft tissues of retroperitoneum). In addition, it is widely known that neoplasms such as EWS may primarily arise in visceral organs. Currently, making a correct diagnosis has become more difficult due to the worldwide use of small incisional biopsies, especially if the tumor shows unusual morphological and immunohistochemical features and/or the pathologist is not familiar with these lesions [2,4,7,8,9]. Accordingly, distinguishing a RMS from an EWS or from the blastematous component of a Wilms’ tumor or from a poorly differentiated NB can be diagnostically challenging, especially if the amount of the material is limited or if the material contains architectural artifacts from sampling and/or if the immunohistochemical results are suboptimal/ambiguous. The diagnosis of SRBCTs, especially on small biopsies, is based on the combination of clinical, radiological, morphological, immunohistochemical and cytogenetic/molecular features [1,2,3,4,5,6]. Although CD99, myogenin and NB84 are considered reliable immunomarkers (highly sensitive but not highly specific) for EWS, RMS and NB, respectively, it is still necessary to identify new immunomarkers for each of the SRBCTs to ensure a correct diagnosis in order to avoid dramatic therapeutic errors [2]. In this regard, the present study confirms that the expression of some immunomarkers can be shared by different SRBCTs: (i) CD99 is expressed by EWS and its T-cell precursor LL; (ii) CD56 is expressed in NB and in the blastemal component of Wilms’ tumor as well as in a minority of cases of alveolar RMS.

Our research group has previously studied the immunohistochemical expression of WT1 and cyclin D1 in the diagnosis of SRBCT on surgically-resected specimens, showing a diffuse and strong cytoplasmic staining for WT1 exclusively in RMS and a diffuse nuclear cyclin D1 expression restricted to EWS and NB [20,32]. The aim of the present study was to validate the utility of these immunomarkers in the differential diagnosis of SRBCTs on small biopsies. We showed that all cases of RMS (33/33), regardless of their subtype, exhibited a strong and diffuse (>90% positive cells) cytoplasmic staining for WT1 (N-terminus). In contrast, neither nuclear nor cytoplasmic immunoreactivity for WT1 was observed in EWS, NB and LL, with the exception of a nuclear positivity in the blastematous and in the epithelial components of Wilms’ tumor. In the present study, five cases of RMS (three embryonal and two alveolar subtypes) which showed only a focal expression of desmin and myogenin along with no staining for MyoD1, were diffusely stained with WT1. Accordingly, this immunomarker could be useful on small biopsies in those cases in which one or two of the three myogenic immunomarkers (desmin, myogenin and MyoD1) are absent or only focally expressed. Although Wilms’ tumor shows nuclear staining with WT1, its diagnosis is usually straightforward if we are dealing with a renal tumor mass with a mixed component (blastemal and epithelial components). However, we admit that serious diagnostic problems might arise if the small biopsy contains solely the blastemal component. As the more recent guidelines for pediatric oncologists indicate that a pediatric renal tumor should undergo chemotherapy for Wilms’ tumor without pre-operative histological diagnosis [41], the possibility that a blastemal-dominant Wilms tumor could be biopsied is very low. However, we advise that any pediatric biopsy, obtained from the retroperitoneum and showing both nuclear and cytoplasmic WT1 positivity (antibodies directed against the N-terminus WT1 protein: clone WT6F-H2) along with CD56 immunostaining, should alert the pathologist to include Wilms’ tumor in the differential diagnosis, especially in the absence of other conventional markers (CD99, NB84, TdT). In this regard we strongly suggest the use of antibodies directed aganist the C-terminus WT1 protein (clone WTC19) which are more specific to Wilms’ tumor (exclusive nuclear staining of the neoplastic blastemal cells). Similarly, a nuclear WT1 immunoreactivity is usually obtained by using anti-C-terminus WT1 protein, as a surrogate of the *EWS-WT1* fusion transcript, in desmoplastic small round cell tumors (DSRCT).

In the present study we also showed that cyclin D1 was diffusely (>50% of neoplastic cells) expressed in all cases of EWS and poorly differentiated NB, while none of the other SRBCT exhibited immunoreactivity for this marker. These results suggest that cyclin D1 is a useful immunomarker in distinguishing EWS and NB from other SRBCTs on small biopsies. This is supported by the fact that cyclin D1 was retained in two cases of EWS which showed both morphological and immunohistochemical artifacts (heterogeneous cellular staining along with non-specific stromal staining with CD99) and in three cases of poorly differentiated NB with absent/focal expression of NB84 (the more sensitive immunomarker for this tumor). As tumors with partial morphological and immunohistochemical overlapping with EWS do exist and have been grouped under the name of “*Ewing-like sarcomas*” [42], including *CIC*-rearranged sarcoma, sarcoma with *BCOR* genetic alterations and round cell sarcoma with *EWSR1*-non-*ETS* fusions, we suggest that any neoplasms with EWS morphology should be tested by molecular analyses for confirming the diagnosis.

In conclusion, we showed that a diffuse and strong cytoplasmic expression of WT1 is of complementary diagnostic utility to myogenic markers (myogenin; MyoD1; desmin) in confirming the diagnosis of RMS (irrespective of subtypes) on small biopsies. Similarly, cyclin D1 can be exploitable as a diagnostic adjunct marker to be used in combination with the conventional immunomarkers CD99 and NB84, to confirm the diagnosis of EWS or NB on small biopsies, respectively. In our opinion, if a tumor histotype, among the wide heterogeneity of SRBCTs, is suspected on a small biopsy, the diagnosis should be confirmed by obtaining the immunoreactivity for at least two or more markers considered sensitive/relatively specific for that tumor, in combination with the absence of any immunoreactivity for other markers. Accordingly, we propose to include WT1 and cyclin D1 in the immunohistochemical panel, along with desmin, myogenin, MyoD1, CD99, NB84, CD56 and TdT for a correct diagnostic approach of pediatric SRBCTs on small biopsies.

## Figures and Tables

**Table 1:** Histotypes and molecular biology profile.

Tumors	Subtypes	Cases Tested by Molecular Biology	Molecular Biology Profile
**RMS** (33 cases)	16 embryonal type 14 alveolar type 3 spindle cell/sclerosing type	10/16 6/14 1/3	0/10 PAX3/FOX01 and PAX7/FOX01 fusions 6/6 PAX3/FOX01 fusion 1/1 MYOD1 L122R mutation
**EWS** (14 cases)	14 classic type	14/14	13/14 EWS/FLI1 fusion 1/14 EWS/ERG fusion
**NB** (44 cases)	44 poorly differentiated	0/44	---
**WT** (3 cases)	3 mixed type	0/3	---
**LL** (11 cases)	7 T-cell precursor 4 B-cell precursor	0/11	---

RMS: rhabdomyosarcoma; EWS: Ewing’s sarcoma; NB: neuroblastoma; WT: Wilms’ tumor; LL: lymphoblastic lymphoma.

**Table 2:** Immunohistochemical results.

Tumors	WT1	Cyclin D1	Desmin	Myogenin	MyoD1	CD99	NB84	CD56	TdT
**RMS** (33 cases)	33/33	0/33	33/33 ^(^*^)^	33/33 ^(^*^)^	28/33	0/33	0/33	3/33	0/33
**EWS** (14 cases)	0/14	14/14	0/14	0/14	0/14	14/14 ^(^**^)^	0/14	0/14	0/14
**NB** (44 cases)	0/44	44/44	0/44	0/44	0/44	0/44	43/44 ^(^***^)^	44/44	0/44
**WT** (3 cases)	3/3	0/3	0/3	0/3	0/3	0/3	0/3	3/3	0/3
**LL** (11 cases)	0/11	0/11	0/11	0/11	0/11	7/11	0/11	0/11	11/11

RMS: rhabdomyosarcoma; EWS: Ewing’s sarcoma; NB: neuroblastoma; WT: Wilms’ tumor; LL: lymphoblastic lymphoma; (*) 5 cases of RMS with focal expression of desmin and myogenin; (**) 2 cases with heterogeneous and extracellular non-specific staining; (***) 2 cases with focal staining.

## Data Availability

All data are available upon reasonable request to corresponding author.
